# Slack resources and individual performance of clinicians: the mediating role of job satisfaction and empirical evidence from public hospitals in Beijing, China

**DOI:** 10.1186/s12913-023-09358-y

**Published:** 2023-04-22

**Authors:** Wei Lu, Xinrui Song, Junli Zhu, Yao Zhang, Changmin Hou

**Affiliations:** 1 School of Public Health, Capital Medical University, Beijing, China; 2 Research Center for Capital Health Management and Policy, Beijing, China; 3 Beijing Jishuitan Hospital, Beijing, China; 4 Beijing Chest Hospital, Capital Medical University, Beijing, China; 5 Beijing Tongren Hospital, Capital Medical University, Beijing, China

**Keywords:** Public hospitals, Clinicians, Slack resources, Individual performance, Job satisfaction, Mediating role

## Abstract

**Background:**

Clinicians in Chinese public hospitals face a complex and severe clinical practice environment, and the individual performance of clinicians is key to improving the output of the healthcare industry. This study aims to explore the mechanism of slack resources in improving individual performance of clinicians and the role of job satisfaction in this process, while the study framework is based on the widely applied Job-Demands Resources theory.

**Methods:**

Based on the study framework composed of slack resources, individual performance, and job satisfaction, hypotheses have been put forward, and questionnaires have been distributed to representative clinicians in tertiary public hospitals. Finally, 318 valid data collected from clinicians have been obtained. To verify the hypotheses, multiple linear regression models have been established to explore the relationship between variables, and the three-stage regression models have been used to verify the presence of mediating role.

**Results:**

All four hypotheses proposed in this study have been proved to be held. Clinicians' job satisfaction has played a mediating role in the impact of slack resources and its three dimensions on individual performance. Among them, there has been a complete mediating role for staff slack, while time and space dimensions have played a partial mediating role in the impact of slack resources on individual performance.

**Conclusions:**

In public hospitals in environments where behavior is subject to significant government interference, it is necessary and feasible to retain appropriate slack resources to improve individual performance. From the perspective of resources management in hospitals, it is necessary for public hospitals to implement a strategy of reserving an appropriate portion of time, staff and space in order to have the conditions to improve clinicians' satisfaction. The existence of slack resources in public hospitals can improve the job satisfaction of clinicians, and then improve the individual performance through the process.

## Introduction

The goals of social organizations are often to provide superior products and services to gain a competitive advantage, while organizations need to improve individual performance to achieve organizational goals [1]. Clinicians are the core group in the provision of medical services in medical institutions such as hospitals, and improving the individual performance of clinicians is increasingly seen as the key to improving the output of the healthcare industry [2]. Individual performance is a major prerequisite for career development and success in the labor market [1], while enterprising clinicians also need to achieving high levels of individual performance.

For the idea of improving employees' job performance, psychologists have proposed some paths, and the most influential theoretical model is the Job-Demands Resources (JD-R) theory, which categorizes the factors affecting employees' job status into two aspects: job demands and resources [3]. The model, which assumes that job requirements and resources produce impaired and motivating processes for job status, respectively [4]. The JD-R theory has been applied more maturely in developed countries in Europe and the United States, for example, Teoh, et al. [5] has used the model perspective as a basis to explore individual and organizational predictors of hospital physician well-being in the United Kingdom, while Kaiser, et al. [6] has used the model to assess Norwegian health practitioners' job demands and job resources as predictors of employee well-being and outcomes factors, and Chênevert, et al. [7] has applied the model to examine the factors underlying Canadian physicians' intention to leave. In China, also Zeng, et al. [8] has applied the model to explore the content of the doctor-patient relationship in Chinese public hospitals, while Zhang, et al. [9] has focused on Chinese physicians as the study group to apply the model.

Person-organization fit theory has believed that individual behavior is the result of the interaction between individuals and their organizational environment, while the level of hospital resources plays an important role as the basic environment for clinicians to provide services. The concept of slack resources derived from the resource-based theory has gradually gained attention [10]. Slack resources and their effects have been widely used in research across industries including healthcare. For example, Valdmanis, et al. [11] has pointed out that the slack resources of health care organizations have been proven to be the cause of differences in service quality and efficiency among health care organizations. Previous literatures have described the concept of slack resources as including “thinking time”, which is particularly important for the improvement of output of healthcare providers as knowledge workers [12]. Job satisfaction is also one of factors related to individual performance, which has been widely studied, while literature has shown that job satisfaction of hospital staff is also one of the organizational performance indicators of hospitals [13]. Niskala, et al. [14] has also pointed out that job satisfaction, which is positively correlated with healthcare service performance, has been critical to the functioning of healthcare organizations. The clinicians’ job satisfaction is a key topic for healthcare managers and patients to focus on. Managers can save on the financial costs associated with high clinician turnover by improving job satisfaction [15], while there is also a positive dynamic relationship between clinicians’ job satisfaction and patient satisfaction [13]. In addition, there is evidence in the field of health care that job satisfaction is related to slack resources of hospitals, while Choi, et al. [16] has pointed out that the staffing and resources of the department can affect the improvement of job satisfaction of nurses.

Among medical institutions in China, public hospitals are in the leading position in the medical system, and the service quantity of public hospitals accounts for about 85% of the total hospital service quantity according to government statistics. China's tertiary public hospitals, which are positioned as medical institutions with functions of medical treatment, teaching, scientific research, and public health services, have been mainly responsible for solving critical illnesses and complex diseases. Public hospitals need highly qualified clinicians who are willing to work efficiently for their institutional goals [17]. Before economic reforms, clinicians have received the same salary regardless of their actual job performance, which has been shown to be inefficient in China. The Chinese government's public hospital reform policy since 2010 has improved the income distribution of clinicians and increased performance-based pay to effectively motivate medical staff [18].

However, the medical reform policies promulgated by the Chinese government have also mentioned that it is forbidden to set income-generating indicators for clinicians, while clinicians' salaries are not linked to the hospital's business income such as medicines and materials [19]. The challenge pressure and hindering pressure of medical staff in public hospitals in China have been significantly higher than those in private hospitals. Chinese clinicians were significantly less satisfied with the organizational environment than nurses and managers, which has also revealed the crowded clinical practice environment in public hospitals [20]. Compared with hospitals of other levels, clinicians in China's tertiary hospitals have high workload, high burnout rate, and high medical mistakes rate, while Wen, et al. [21] has also shown that about 80% suffer from burnout and 60% report having made medical mistakes in the past year. In public hospitals, it is necessary to explore ways to improve clinicians' individual performance, so this study has focused on the public hospitals in China as the place for investigation. At present, only Chen, et al. [22] has showed that high-performance systems can affect performance through resources and burnout through job demands, while there is a gap in research exploring this issue from the slack resources perspective, so this study would be proposed.

## Methods

### Study framework and hypotheses

This study constructs a research framework based on Job-Demands Resources theory, which has been initially widely used in manufacturing, finance, and transportation, among others [4], and has been widely used in hospital and physician management-related research in recent years. Previous literatures have all applied Job-Demands Resources theory from multiple perspectives to study the topic of hospitals and physicians, but there are two main shortcomings: one is the neglect of the interaction between job demands and resources, and the other is the lack of in-depth exploration of the specificities of the hospitals. Based on the defining criteria of Van, et al. [4], job demands refer to workload and clashes, and job resources refer to the support received at working process, while some empirical studies based on this theory have existed in recent years. In recent years, some scholars have emphasized that the interaction of job demands and job resources has a significant impact on job status [23], while this study has been inspired from the model by substituting slack resources and job satisfaction into the model's job resources and requirements, respectively, to explore the relationship between the two and their interactions with clinicians' performance. Next, the definitions of physicians' individual performance, slack resources and job satisfaction in this study will be presented to distinguish them from other studies.

Individual performance is the record of achievement produced by the function of a staff to perform a specific job or activity during a specific work period [24]. Khan, et al. [25] has defined individual performance as work performance in terms of quantity and quality expected from each staff. Gould-Williams & Davies [26] has believed that individual performance is the most important work outcome and a key factor affecting organizational performance, success and competitiveness. The new medical reform policies in China have defined that the individual performance of clinicians should reflect the ability, quality, effectiveness, and efficiency of the medical care services they provides.

The concept of slack originated in organizational theory, which has been explicitly proposed by Cybert & March [10], while Nohria & Gulati [27] has defined slack resources to exceed the minimum resources required to produce given to organizations. Slack resources have been considered as the buffer resources that enables an organization to successfully adapt to changes in internal and external pressures, while the study of Mallidou, et al. (2011) and Chamberlain (2016) based on healthcare industry have divided slack into three dimensions including time, staff and space [12, 28].

Job satisfaction refers to the general attitude of staffs towards their jobs, describing how happy or unhappy, satisfied or dissatisfied they feel at work [29]. Andersen, et al. [30] has defined job satisfaction as a pleasant emotional state resulting from meeting or exceeding expected work outcomes. The most accepted definition of job satisfaction has come from Locke [31], which has defined job satisfaction as a positive emotional feeling that results from a staff's evaluation of his job by comparing his expectations with what he actually thinks it provides or needs. Ostroff [32] has said that job satisfaction is directly related to individual needs, including challenging work, fair rewards, supportive job surrounding and colleagues.

The classic view in 1959 based on resource-based theory has held that slack resources is more important as a driving force for performance growth than the total resources, while there is still no unified understanding of the impact of slack resources on performance. Wiersma [33] has argued that slack resources are neither beneficial nor harmful, but the use of it by managers determines whether slack resources play an advantageous or disadvantageous role. Zhong [34] has indicated that the level of slack resources in organizations is closely related to the development of the organization, at least slack resources should be regarded as a benign benefits of staffs to improve individual performance level. Guo, et al. [35] has found that slack resources provide conditions for individuals to promote work output by focusing the slack resources on the expected goals. According to the above, this study proposes the following hypothesis:

- *H1(a/b/c): Slack resources(Time/Staff/Space) and individual performance have a positive correlation.*

According to situational theories, job satisfaction arises from various aspects of the nature and environment of job [36], and slack resources have been regarded as one aspect of job environment. Jungyoon, et al. [37] pointed out that characteristics of resources possessed by the organizational environment such as information availability and feedback mechanisms are related to job satisfaction, while Baloc, et al. [38] has pointed out that the amount of resources in the workplace is positively correlated with job satisfaction, and staffs with higher resources levels would show higher levels of satisfaction compared to those with lower resources levels. Similar evidence has existed in the field of health care (primarily nursing), Chamberlain, et al. [28] demonstrated through an empirical study of nursing assistants that slack in staff and time dimensions is associated with increased job satisfaction, while their other study has found that nursing assistants' perception of lower space slack led to lower work enthusiasm [39]. Accordingly, the following hypothesis can be made:

- *H2(a/b/c): Slack resources(Time/Staff/Space) and job satisfaction have a positive correlation.*

The relationship between individual performance and job satisfaction is one of the most studied topics in the field of organizational management, while one of the more classic propositions is "Happy workers are productive workers". This view has originated from human relations theory, and Andersen, et al. [30] has pointed out that the proposition of a causal relationship between job satisfaction and individual performance can be traced back to the Hawthorne study, but the nature of this relationship has also remained controversial in recent years. Edwards, et al. [40] has used social cognitive theory to explain the relationship between job satisfaction and performance, and has proposed that staffs' attitudes towards work would affect their behavior at work, and their behavior would also have effects on the formation of their attitudes. However, the impact of job satisfaction on individual performance has been demonstrated in multiple fields. For example, Jusmin [29], which have taken lecturers as research subjects, has found that job satisfaction has a significant positive effect on individual performance. And Atmojo [41] also has pointed out that job satisfaction has a significant positive effect on performance based on managers in state-owned enterprises. Satisfied staffs are highly motivated to work harder and ultimately tend to achieve superior individual performance, while this study can make the following hypothesis:

- *H3: Job satisfaction and individual performance have a positive correlation.*

**Fig. 1:**
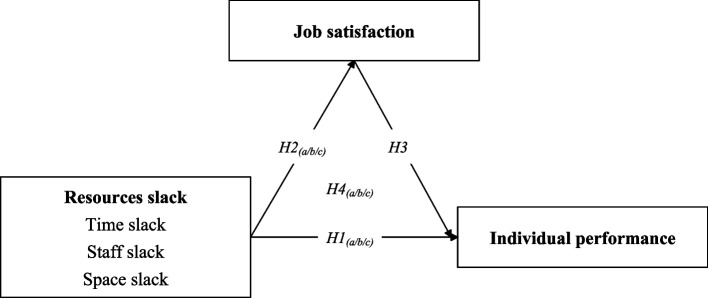
Study framework

Based on the review of previous literature, this research under the framework of behavioral decision theory, starting from basic concepts, proposes hypotheses and conducts empirical verification along the path of problem search, response and solution. The study framework is shown in Fig. 1, while this study can make the following hypothesis based on all of the above evidence.

- *H4(a/b/c): Job satisfaction plays a mediating role in the positive correlation between slack resources (time/staff/space) and individual performance.*

### Sample and data

This study uses the questionnaire survey to collect the required relevant information, and relies on the electronic system to publish the questionnaire online. This study selects clinicians in tertiary public hospitals as the respondents. The clinical group has a more accurate perception of slack resources, and their satisfaction and job performance are related to the operation and development of the hospital's core services. The sample size required for this study was calculated based on the sample size formula and was initially estimated to be about 260 cases, which was increased by 20–30% on top of that taking into account factors such as refusal to visit, and was finally determined to be about 320 cases. In this study, multi-stage sampling was used to first divide the hospitals in Beijing into two strata: general hospitals and specialist hospitals, and six general hospitals and five specialist hospitals were randomly selected according to the optimal allocation stratification. According to the total number of clinicians in each hospital, the sampling proportion and number of samples in each target hospital has been determined, and a sufficient number of respondents has been randomly recruited in each hospital. The questionnaires have been distributed for three months in 2021, and the distribution venues are 6 general tertiary public hospitals and 5 specialized tertiary public hospitals. Finally, a total of 318 questionnaires filled out by clinicians have been obtained. Online recruitment was conducted within the hospital relying on electronic systems and local area networks, and all respondents included signed informed consent forms. All respondents have participated in the study voluntarily and provided information anonymously to ensure that the information quality, while the collected data does not contain any identifiable information. This study was approved by the Medical Ethics Committee of Capital Medical University.

### Instruments

The content of the questionnaire includes two parts: basic information and scales. The basic information is the characteristics of hospitals and individuals, and the scales in the questionnaire have been scored on a 7-point Likert scale, with options ranging from completely disagree/very dissatisfied to completely agree/very satisfied. The scales selected were all well-established scales with good reliability and validity used in the previous literature, and no unauthorized additions or deletions were made to the scales entries in this study. To ensure the rationality of scale selection, this study has taken a series of measures to improve quality. Firstly, scales have been translated from English to Chinese, while some expressions have replaced according to the topic of this study. Secondly, a group of five experts has been formed to discuss the modification of items. Thirdly, one hospital has been selected to conduct a small sample pre-survey before formal survey.

The slack resources scale used in this study has been derived from Anastasia,et al. [12], with 9 items in total. The scale has divided slack resources of hospitals into three dimensions: time slack (4 items), staff slack (2 items), and space slack (3 items), with good reliability (Cronbach's α is 0.74, 0.83, 0.63, respectively). Respondents have answered questions such as "Does your department have enough staff to get the job done". In this study, the scale has also showed high reliability (Cronbach's α is 0.87, while the three subscales are 0.74, 0.90, 0.89).

The individual performance scale used in this study has referred to the 5-item scale of Podsakoff & Mackenzie [42]. Respondents have answered questions such as "Are you able to meet all job performance requirements and achieve performance goals". This scale applies to a variety of industries including healthcare, while Cronbach's α is 0.602 in this study.

The job satisfaction scale used in this study has come from Schriesheim & Tsui [43] which has 6 items, while Cohen [44] has used the above scale with good reliability to conduct staffs of public organizations. Respondents have answered questions such as "How satisfied I am with the nature of the work I do" and "How satisfied I am with the relationships between colleagues in the hospital". Cronbach’s α coefficient is 0.77 in Cohen [44], compared to 0.887 in this study.

In this study, two types of variables have been selected as control variables by consulting relevant literature and combining with the actual situation in China. The first category is hospital characteristics including nature, beds, location, and number of colleagues, while the second category is personal characteristics including sex, age, job title, years of employment, and working hours per week.

### Statistical analysis

Stata 16.0 statistical software has been used to analyze the data. Quantitative data has been described using mean and standard deviation, while qualitative data has been described using frequency and percentage description. Then, this study has performed correlation analysis to obtain preliminary relationships between variables. This study has proposed to construct the three-stage hierarchical regression models because regression analysis is more advantageous for revealing the specific paths of action among variables in the model [45], while the method is more mature and avoids problems such as measurement errors that undermine statistical significance and multicollinearity [46]. To verify the four conditions of the mediation hypothesis, multiple linear regression models have been established to explore the relationship between variables. The relationship of variables in the regression models is as follows:

1 $$"Individual\;Performance"\:=\:\beta_{11}\;"Slack\;Resources"+\;\beta_{01}$$

2 $$"Job\;Satisfaction"\:=\:\beta_{12}\;"Slack\;Resources"\;+\;\beta_{02}$$

3 $$"Individual\;Performance"\:=\:\beta_{13}\;"Job\;Satisfaction"+\;\beta_{03}$$

4 $$"Individual\;Performance"\:=\:\beta_{14}\;"Slack\;Resources"\:+\:\beta_{24}\;"Job\;Satisfaction"\:+\:\beta_{04}$$

If the regression coefficient *β*_*11*_ ~ *β*_*13*_ of the variable of Eqs. (1) ~ (3) is significant, and the regression coefficient *β*_*24*_ of the intermediate variable of Eq. (4) is significant, but *β*_*14*_ is not significant (or significant but *β*_*14*_ is less than *β*_*12*_), it can be considered that job satisfaction has a mediating role in the positive correlation between slack resources and individual performance. If *β*_*14*_ is not significant at all, it indicates that job satisfaction has played completely mediating role, while if *β*_*14*_ is only reduced but still significant, it is only partial mediating role.

## Results

### Descriptive research and correlation analysis

Basic characteristics of the sample has been shown in Table 1. As can be seen from the description, 50.94% of the respondents come from general hospitals, while most are from hospitals with 1,000 beds or more (61.64%). 56.92% of the respondents are female, with an average age of 34.16 years. Respondents have an average of 7.46 years in medical work, generally working an average of 55.80 h per week. Scores of scales and correlation analysis have been shown in Table 2. Checking the correlation coefficient matrix of variables can be considered that there is almost no severe multicollinearity in the regression models, while Harman's Single-Factor Test was applied to the scale and it was found that none of the variance explained by the unrotated first factor exceeded 40%, which could exclude the effect of common bias on the study.

**Table 1:** Descriptive statistics of basic characteristics

Hospital variables	N(%)
Hospital nature	
General hospital	162 (50.94)
Specialist hospital	156 (49.06)
Hospital beds	
Below 1000 beds	122 (38.36)
1000 beds or more	196 (61.64)
Hospital location	
Core functional	211 (66.35)
Function expansion	107 (33.65)
Number of co-workers	
≤ 10	65 (20.44)
11 ~ 20	90 (28.30)
21 ~ 50	84 (26.42)
51 ~ 100	43 (13.52)
> 100	36 (11.32)
Personal variables	N(%) / M ± SD
Sex	
Female	181 (56.92)
Male	137 (43.08)
Age	34.16 ± 6.96
Job title	
Senior	51 (16.04)
Intermediate	124 (38.99)
Junior and below	143 (44.97)
Working years	7.46 ± 6.54
Working hours per week	55.80 ± 14.33

**Table 2:** Scores of scales and correlation analysis

Variables	M ± SD	Correlations				
		Individual performance	Slack resources	Time slack	Staff slack	Space slack
Individual performance	24.63 ± 4.47	1				
Slack resources	36.83 ± 10.70	0.354	1			
Time slack	17.86 ± 4.76	0.307	/	1		
Staff slack	7.84 ± 3.42	0.232	/	0.376	1	
Space slack	11.13 ± 4.95	0.311	/	0.449	0.657	1
Job satisfaction	29.14 ± 7.01	0.525	0.493	0.396	0.377	0.424

### Regression models based on the overall slack resources level

The regression model of the relationships between slack resources and individual performance is shown in Table 3. From Model 1, it can be found that the variable of slack resources is significant, which confirms that *H2* holds. Model 2 shows the relationships between various control variables and individual performance, among which the working years is a significant variable. Model 3 uses time slack, staff slack, and space slack as independent variables simultaneously, of which time slack and space slack are significant factors (*p* < 0.01), and staff slack is not significant. Model 4 uses job satisfaction as an independent variable and finds that it is statistically significant (*p* < 0.01), which confirms that *H3* holds. Model 5 uses the overall slack resources level as an independent variable, which is confirmed as a significant factor (*p* < 0.01), while this confirms that *H1* holds. From Model 6, it can be considered that job satisfaction has a mediating role in the positive correlation between the overall slack resources level and individual performance. Compared with model 5, the significance of the overall slack resources level is weakened (*p* < 0.05) and the coefficient is also greatly reduced, while job satisfaction is more significant (*p* < 0.01) and H4 is confirmed to hold.

**Table 3:** Regression model of the overall slack resources level

Variables	Model1^α^	Model2^β^	Model3^β^	Model4^β^	Model5^β^	Model6^β^
Slack resources	0.334^***^				0.152^***^	0.0605^**^
Time slack			0.164^***^			
Staff slack			0.0292			
Space slack			0.219^***^			
Job satisfaction				0.320^***^		0.273^***^
Hospital nature	4.585^***^	1.251	2.245^***^	0.422	2.157^***^	0.905
Hospital beds	1.251	-0.0822	0.748	0.0239	0.636	0.295
Hospital location	-1.225	0.319	-0.224	0.351	-0.192	0.142
Sex	-0.768	-0.439	-0.679	-0.393	-0.722	-0.512
Age	0.244^**^	0.122	0.120	0.0432	0.121	0.0543
Working years	-0.364^***^	-0.178^**^	-0.140^*^	-0.0338	-0.139^*^	-0.0395
Job title	-0.709	-0.835	-0.514	-0.349	-0.468	-0.274
Working hours per week	0.00729	-0.00101	0.00991	0.00539	0.0114	0.00939
Staff in department	-0.0597	-0.101	-0.106	-0.103	-0.131	-0.115
Constant	10.43^**^	23.59^***^	15.78^***^	14.73^***^	15.76^***^	12.91^***^
*R*^2^	0.323	0.061	0.187	0.292	0.182	0.306
Adjusted *R*^2^	0.301	0.033	0.155	0.269	0.155	0.281

*N* = 318, ^α^ Job satisfaction as dependent variable,^β^ Individual performance as dependent variable, ^*^p < 0.1, ^**^p < 0.05, ^***^p < 0.01

### Regression models based on the time, staff and space slack level

Take time slack, staff slack, and space slack as independent variables, respectively, and the models are shown in Table 4. In order to verify the mediating role of job satisfaction, Model 1 uses job satisfaction as the dependent variable and three dimensions of slack are included in the three models as independent variables, which are all significant (*p* < 0.01). *H2* is confirmed to hold by this model. It can be seen from Model 2 that there is a significant relationship between job satisfaction and individual performance (*p* < 0.01), while Model 3 includes three dimensions of slack into the models and they are all significantly related to individual performance (*p* < 0.01), which confirms the holding of *H3* and *H1*. Model 4 takes job satisfaction and three types of slack (respectively) into the models simultaneously. Among them, job satisfaction is a significant factor in each model (*p* < 0.01), time slack and space slack still significantly related to individual performance (*p* < 0.05), but the regression coefficients are significantly lower than model 3, and staff slack is not significant. The above results show the mediating role of job satisfaction, which has a partial mediating role on the positive relationships between time slack or space slack and individual performance, and a complete mediating role on staff slack. In summary, it can be assumed that *H4* holds.

**Table 4:** Regression model based on the time, staff and space slack level

Variables	Model1^α^			Model2^β^	Model3^β^			Model4^β^		
	(1)	(2)	(3)		(1)	(2)	(3)	(1)	(2)	(3)
Time slack	0.551^***^				0.268^***^			0.108^**^		
Staff slack		0.795^***^				0.314^***^			0.0696	
Space slack			0.656^***^				0.303^***^			0.119^**^
Job satisfaction				0.320^***^				0.292^***^	0.307^***^	0.282^***^
Hospital nature	2.874^**^	3.781^***^	5.187^***^	0.422	1.389^*^	1.720^**^	2.453^***^	0.551	0.561	0.990
Hospital beds	0.156	0.328	1.650	0.0239	0.155	0.178	0.835	0.110	0.0772	0.369
Hospital location	-0.605	-0.704	-1.208	0.351	0.0727	0.0806	-0.194	0.249	0.297	0.147
Sex	-0.108	-0.863	-0.820	-0.393	-0.421	-0.722	-0.751	-0.389	-0.457	-0.520
Age	0.255^**^	0.244^**^	0.234^**^	0.0432	0.126	0.121	0.116	0.0517	0.0462	0.0503
Working years	-0.390^***^	-0.400^***^	-0.395^***^	-0.0338	-0.149^*^	-0.158^**^	-0.152^**^	-0.0349	-0.0354	-0.0409
Job title	-0.842	-0.924	-1.225^*^	-0.349	-0.506	-0.601	-0.699	-0.260	-0.317	-0.354
Working hours per week	-0.00953	0.00477	0.000661	0.00539	0.00409	0.00876	0.00855	0.00687	0.00729	0.00836
Staff in department	-0.0489	-0.134	0.0577	-0.103	-0.128	-0.157	-0.0776	-0.114	-0.116	-0.0938
Constant	14.86^***^	18.44^***^	16.70^***^	14.73^***^	17.35^***^	19.95^***^	18.51^***^	13.02^***^	14.29^***^	13.80^***^
*R*^2^	0.219	0.225	0.278	0.292	0.139	0.114	0.163	0.303	0.294	0.304
Adjusted *R*^2^	0.193	0.200	0.254	0.269	0.111	0.086	0.135	0.278	0.269	0.279

*N* = 318, ^α^ Job satisfaction as dependent variable,^β^ Individual performance as dependent variable, ^*^p < 0.1, ^**^p < 0.05, ^***^p < 0.01

## Discussion

This study has mainly discussed the process in public hospitals of making better use of various slack resources to promote clinicians' individual performance by improving clinicians' job satisfaction. A series of hypotheses have been proposed based on the Job-Demands Resources theory, and the hypotheses have been supported by empirical evidence from the results of this study.

The results of the regression models have suggested that *H1* holds at both the aggregate level and the three-dimensional level. There has been a significant positive correlation between slack resources in public hospitals and individual performance of clinicians, while the mechanism can be discussed as follows. Early studies such as Jensen & Meckling [47] has once believed that enterprises' slack resources are wasteful and inefficient due to managers' selfish interests. Zahargier & Balasundaram [48] has pointed out that individual performance may have different effects depending on the conditions and things of job surrounding, which includes slack resources as a very important aspect. Ibrahim & Yusra [49] has argued that when the job surrounding meets the needs of staff, they will align their efforts with their job surrounding, while a moderate amount of slack resources can enable staff to actively pursue goals, which may lead to improvements in their performance [34]. Marlin [50] has pointed out that the level of various slack resources in the medical industry is higher than that in other industries. In public hospitals, the slack medical resources will also make the work of medical staff more malleable and possible, such as providing patients with more convenient and efficient clinical treatment plans, so that individual performance will be improved. This can be verified by the study of Mallidou, et al. [12], which has pointed out that slack resources in hospitals may allow clinicians to use the extra time and space to develop or implement innovative behaviors that improve individual performance when used in daily work. From the above, hospital managers have reasons to set time, staff, and space slack for the hospital to better promote the performance of clinicians.

This study has confirmed that job satisfaction mediates the role of slack resources on individual performance. That is, *H4* as shown in the regression models hold. Since satisfaction is generated from the heart of the staff, the role of staff slack is fully mediated by satisfaction, while slack in time and space dimensions is only partially mediated. Shafique, et al. [51] has used job satisfaction as a mediating variable to explain the relationship between certain environmental characteristics of organizations and individual performance, while Graham [52] has shown that promoting a psychologically satisfying workforce may be a means of improving individual performance if staffs have positive perceptions of the characteristics of the job surrounding. Similar little evidence has existed in the healthcare industry. Lowe [53] has found that staffs in the health care industry have low evaluation of the level of slack resources of time, individual, and space in their institutions, while only 20%-40% of staff have believed that the resources have been sufficient, which may have resulted in lower outcome output. Han, et al. [54] has proposed that hospitals in China should create a good resources support system for clinicians to strengthen the sense of belonging of staff, so that doctors are willing to engage in behaviors beneficial to the hospital, thereby improving individual performance. The results of this study have been a good complement to the above research, while the following two aspects will introduce the mediating process of job satisfaction.

The *H2* confirmed by the regression models hold, further emphasizing the importance of slack resources. In this study, the allocation level of hospital slack resources has been significantly positively correlated with clinicians' job satisfaction, while slack resources in the three dimensions of time, staff, and space are all beneficial to the improvement of job satisfaction. The results of this study on time slack have supported the findings of Probst, et al. [55], which has said that medical staffs’ job satisfaction would decrease when they feel that they do not have enough time to complete their tasks. In addition, this study has also confirmed that the existence of staff slack is related to the improvement of job satisfaction, similar to the conclusions of Chamberlain, et al. [28], which has pointed out that front-line health care staffs often hope to increase staffing. And Aloisio [56] has pointed out that there has been a correlation between nurses' job satisfaction and perceptions of organizational space availability, and the availability of slack resources has required to achieve organizational goals is important for improving job satisfaction.

In this study, clinicians' job satisfaction has contributed to improved individual performance, while the holds of *H3* in this study is also supported by the results of regression models. Previous studies have also dissected this mechanism such as Mirzaii, et al. [57], which has pointed out that job satisfaction is critical to success in work and increases motivation levels to lead to higher levels of efficiency. In the education industry, Genelyn, et al. [58] has pointed out that the supervision and restriction of teachers' work are not conducive to the improvement of their individual performance, but their satisfaction with the work itself and the relationship with colleagues is beneficial to the improvement of individual performance. While the evidence in the field of the healthcare industry has been certainly no exception. Because clinicians have slack time and space to exert their subjective initiative, it is easy to produce a pleasant emotional state, stimulate self-efficacy, and motivate more work performance. Ikyanyon & Ucho [59] has studied staff of a federal hospital in Nigeria and has found that staff with high job satisfaction achieved better individual performance than those with low satisfaction, which is similar to the results obtained in this study.

This study has some contributions. From the theoretical perspective, this study has applied Job-Demands Resources theory to the study of individual performance of hospital physicians and has focused on exploring the interaction between two major aspects, and tentatively hypothesizes that job satisfaction may have a mediating role in the relationship between slack resources and individual performance, contributing to a broader extrapolation of the theory for use. From the practical perspective, this study has the following three contributions. First, this study has taken public hospitals as the research object, making up for the lack of research on the impact of slack resources in the medical industry on individual performance. And unlike most studies of nurses or managers [28, 55], this study provides evidence from clinicians. Second, unlike most studies based on clinicians in developed countries [17], the location of this study is China, which has a special political environment. Public hospitals in China are in a highly politicized environment that is subject to significant government interference, while the evidence in this environment is presented in this study. Third, based on previous theories and experience from other industries, this study has selected clinicians’ job satisfaction as a mediator of the relationship between slack resources and individual performance, which has certain implications in the hospital management field.

This study also has some limitations, which may be resolved in future studies. First, this study is a cross-sectional design with limited causal linkages, while the results cannot reflect the process of increasing and decreasing the impact of slack resources on clinicians. And longitudinal time-series design models can be used in future studies. Second, the measurement of performance and slack resources in this study has been subjective reflections of the clinicians. Self-evaluations are often less accurate than peers' or supervisors' evaluations compared to objective standard measures. The use of uniform objective criteria to measure clinician performance and hospitals’ slack resources can be considered in future research. Third, the group of clinicians selected in this study is narrow, and only tertiary public hospitals in Beijing have been investigated, while clinicians from other levels and types of hospitals may be considered for selection in future research.

## Conclusions

Based on the review of previous literature, this study has conducted a cross-sectional survey with clinicians in Chinese public tertiary hospitals as the research objects. Based on the theoretical framework of Job-Demands Resources theory, some hypotheses are proposed and supported by empirical studies. This study has confirmed the relationships between hospital slack resources, clinicians' individual performance and job satisfaction, while this study has found that job satisfaction mediates the relationship between slack resources and individual performance. This study not only broadens the application of Job-Demands Resources theory, but also highlights the importance of slack resources and has certain reference value for managers in hospitals to better allocate health resources to clinicians to improve performance.

## Data Availability

The datasets used and analysed during the current study are available from the corresponding author on reasonable request.
